# S230. LONG-TERM ANTIPSYCHOTIC MEDICATION IN SCHIZOPHRENIA: BENEFITS, RISKS AND FOLLOW-UP: DATA FROM FINNISH COHORT STUDIES AND SYSTEMATIC REVIEW

**DOI:** 10.1093/schbul/sby018.1017

**Published:** 2018-04-01

**Authors:** Matti Isohanni, Jouko Miettunen, Erika Jääskeläinen, Jani Moilanen, Anja Hulkko, Sanna Huhtaniska

**Affiliations:** University of Oulu

## Abstract

**Background:**

Millions of people use antipsychotic medications. Thousands of clinicians (often non-psychiatrists) prescribe and monitor them every day. Existing research reports mostly favorable risk-benefit ratio during the first years of schizophrenia, but their risk-benefit ratio and maintenance efficacy in long-term is not clear.

Our aim was to:

1. analyze long-term antipsychotic use and its determinants in Finnish cohort samples, and

2. review the studies on benefits, risks, and follow-up and monitoring practices of long-term antipsychotic treatments.

**Methods:**

1. We used the data of population-based Northern Finland Birth Cohort 1966 (NFBC1966), and also Finnish therapeutic community data.

2. We performed a systematic literature search on long-term treatment effects, risks and monitoring of antipsychotic medication in schizophrenia.

**Results:**

1. In NFBC1966 in midlife, higher lifetime doses of antipsychotics were associated with alterations in brain morphometry, poorer neurocognition, and poorer clinical outcomes. Clinical follow-up was inadequate even in half of the schizophrenia cases. In therapeutic community cohort, maximal development of psychosocial care reduced the mean dose of antipsychotics in acute psychosis ward from 370 mg/day as chlorpromazine equivalents into 160 mg/day. 2. In the literature review, three main cornerstones in the high quality longitudinal use of antipsychotic medication were: a) high, evidence-based pharmacological quality, b) optimal adjuvant psychosocial therapies, c) sophisticated long-term prescription, monitoring and follow-up practices to minimize nonadherence and psychiatric and somatic failures.

In sum, antipsychotics are effective for acute and mid-term psychosis in prevention of relapses and excess mortality. Long term antipsychotic use especially in high doses may include major iatrogenic harms, as also poorly monitored withholding or discontinuing. When aiming for an optimal benefit-risk ratio and for balancing symptomatic, functional and somatic outcomes, the goal is to aim for lower ranges of effective dosing, as well as choosing an appropriate antipsychotic agent that causes minimal side effects, and to combine adjuvant psychosocial interventions in the treatment. The often recommended personalized smallest effective dose is not so simple but still a realistic strategy in current relapse prevention practices, where doses often are too large for safety reasons.

**Discussion:**

Cohort-based register studies are useful in examining long-term medication effects although they contain a risk of residual confounding due to their observational design. However, randomized controlled trials in long, over 3–7 years of follow-up, are unrealistic.

The systematic literature review demonstrates major open or conflicting questions in risk-benefit ratio related to long-term outcomes. Non-adherence and attrition are key problems in sustained antipsychotic medication. Standardized prescription and monitoring practices (not so much studied) might improve medication adherence and also outcomes. Current clinical guidelines advise us based on studies from first years of schizophrenia. There are only few and weak patient-level predictors of successful tapering and discontinuation of antipsychotic medication.

In the future, clinical follow-up of medication can be improved by structured follow-up and planned continuity. Life span view of antipsychotic medication stresses careful documentation of doses, responses and harms, longitudinal planning and realization of medication as part of the whole treatment program, as well as individualized and tailored selection, dosing (dose as low as possible or minimal effective dose) and follow-up by a well-trained team.

